# Multi-Scale Understanding of NMDA Receptor Function in Schizophrenia

**DOI:** 10.3390/biom10081172

**Published:** 2020-08-11

**Authors:** Jo Soo Hyun, Takafumi Inoue, Akiko Hayashi-Takagi

**Affiliations:** 1 Laboratory for Multi-scale Biological Psychiatry, Center for Brain Science, RIKEN, 2-1 Hirosawa, Wako City, Saitama Prefecture 351-0106, Japan; soohyunjo@hotmail.com; 2 Department of Life Science and Medical Bioscience, School of Advanced Science and Engineering, Waseda University, Tokyo 162-8480, Japan; inoue.t@waseda.jp

**Keywords:** multi-scale, synaptic pathology, schizophrenia, synaptic optogenetics

## Abstract

Schizophrenia is a chronic and disabling psychiatric disorder characterized by disturbances of thought, cognition, and behavior. Despite massive research efforts to date, the etiology and pathophysiology of schizophrenia remain largely unknown. The difficulty of brain research is largely a result of complex interactions between contributory factors at different scales: susceptible gene variants (molecular scale), synaptopathies (synaptic, dendritic, and cell scales), and alterations in neuronal circuits (circuit scale), which together result in behavioral manifestations (individual scale). It is likely that each scale affects the others, from the microscale to the mesoscale to the macroscale, and vice versa. Thus, to consider the intricate complexity of schizophrenia across multiple layers, we introduce a multi-scale, hierarchical view of the nature of this disorder, focusing especially on *N*-methyl-D-aspartate-type glutamate receptors (NMDARs). The reason for placing emphasis on NMDAR is its clinical relevance to schizophrenia, as well as its diverse functions in neurons, including the robust supralinear synaptic integration provided by *N*-methyl-D-aspartate-type glutamate (NMDA) spikes and the Ca^2+^ permeability of the NMDAR, which facilitates synaptic plasticity via various calcium-dependent proteins. Here, we review recent evidence implicating NMDARs in the pathophysiology of schizophrenia from the multi-scale perspective. We also discuss recent advances from optical techniques, which provide a powerful tool for uncovering the mechanisms of NMDAR synaptic pathology and their relationships, with subsequent behavioral manifestations.

## 1. Introduction

**Figure 1:**
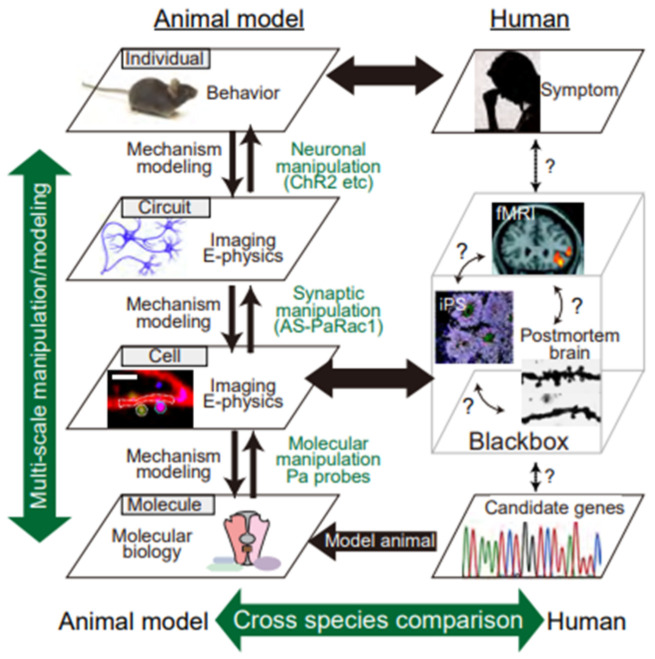
Multi-scale, hierarchical nature of psychiatric disorders. Despite extensive recent efforts, the pathogenesis of schizophrenia remains poorly understood, mainly because its pathophysiology results from synergistic interactions between variants of multiple genes and environmental factors. Thus, the known contributory factors for this disease are susceptible gene variants (molecular layer: genetic studies, etc.), synaptopathies (subcellular and cell layer: iPS and postmortem studies, etc.), and alterations in neuronal circuits (circuit layer: brain imaging, such as fMRI, etc.), conceivably resulting in behavioral manifestations (individual layer: symptomatology). However, human studies have been limited to single layers, which hinders the integrative and causal mechanistic understanding of behaviors. Therefore, we must deal with the intricate complexity of behaviors in schizophrenia-related animal models. Multi-scale analysis consists of an electrophysiological method and Ca^2+^ imaging to visualize the synaptic input (synaptic level), dendritic events (dendritic level), action potentials (cell level), and behavioral manifestations (individual level). In addition, with the use of in vivo optical and in silico manipulation of molecules, synapses, and cells in animal models of schizophrenia, researchers can determine the type of pathology that underlies the disorder in the animal model.

Schizophrenia is a chronic and devastating mental illness. Despite rapid advancements in various state-of-the-art technologies, neuroscience still cannot provide a curative treatment for schizophrenia, largely because of a lack of precise understanding of the pathophysiology of the disorder. This is primarily due to the challenge of bridging the large-scale gap, i.e., the gap between the nano- and micro-scales, where chemical and molecular principles govern, and the much larger macroscale of the symptomatic phenomena we wish to understand (Figure 1). Previous genetic studies have demonstrated that schizophrenia is a highly heritable disorder [1,2,3]. However, genetic association does not provide insights into the network of causality between the synapse, cell, circuit, and behavioral scales of the disorder. Thus, no matter how significantly associated a genetic variant is with the disorder, it remains unclear which variant of which gene should be studied to dissect disease-related mechanistic components. To develop medical treatments, an understanding of the pathology mechanism is essential; this includes the specific molecular targets to which the drug binds, such as enzymes, transporters, or receptors. Therefore, as long as we are focusing on a single scale of the brain at a time, available research strategies remain limited [4]. In this review, we will introduce the advantages of multi-scale studies (i.e., studies of inter-scale interactions) using animal models, with the goal of examining how the distinct scales of the brain interconnect, ultimately determining behavioral alterations in the context of brain disorders (Figure 1). In particular, in light of the accumulating evidence mentioned below, we will focus on the multi-scale aspect of *N*-methy1-d-aspartate receptor (NMDAR) signaling as a potential contributing factor for schizophrenia. Multiple studies have concluded that schizophrenia can, at least in part, be considered as an NMDAR disorder. However, due to the inaccessibility to the living human brain at synaptic resolution, the detailed biological alterations of NMDARs across multiples scales remain almost entirely unknown. Hence, we will discuss tools for multi-scale investigation, including animal models and newly developed optogenetic techniques.

## 2. Functions of NMDAR Signaling

**Figure 2:**
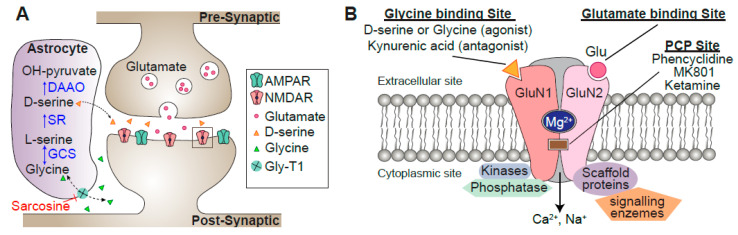
Schematics of a dendritic spine. (**A**) Activation of N-methyl-D-aspartate-type glutamate receptors (NMDARs) requires glutamate and the co-agonist D-serine or glycine. Synaptic NMDARs have preferential affinity for D-serine, and extrasynaptic NMDARs for glycine. In astrocytes, glycine imported by Gly-T1 is metabolized by the glycine cleavage system (GCS). Serine racemase (SR) converts L-serine into D-serine, which is released from astrocytes through exocytosis following activation of receptors on the astrocyte plasma membrane. Once in the synaptic cleft, D-serine binds to synaptic GluN2A subunits. (**B**) Schematic of the NMDAR structure with its main binding sites. Mg^2+^ binds to a specific site on the receptor, blocking the passage of cations until the postsynaptic membrane is sufficiently depolarized. GluN2 also has a binding site for several psychomimetic open-channel blockers, such as phencyclidine (PCP) and ketamine. Scaffolding proteins in the postsynaptic density assemble various kinases, phosphatases, and enzymes close to their substrates, connecting NMDARs to the molecules involved in signal transduction, the cytoskeleton, and receptor clustering.

**Figure 3:**
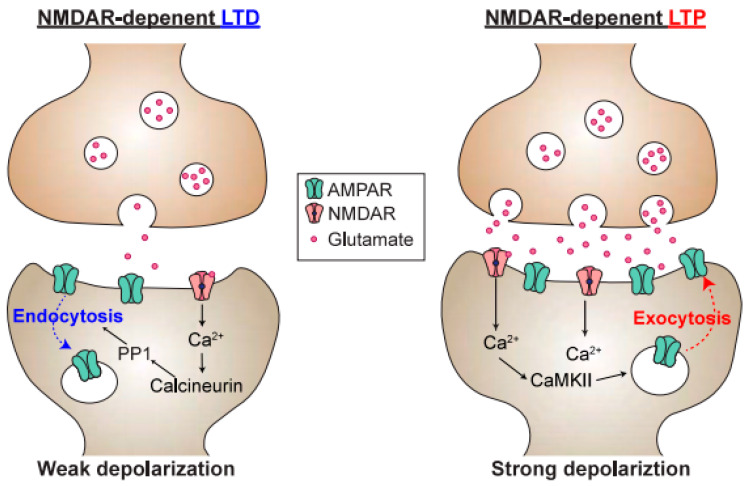
Schematic of a synapse undergoing long-term depression (LTD) or long-term potentiation (LTP). Ca^2+^ entry through NMDARs is responsible for both postsynaptic LTD and LTP, and postsynaptic Ca^2+^ concentration is a determinant of whether the synapse undergoes LTD or LTP. Because calcineurin has a much higher affinity for Ca^2+^/Calmodulin than CaMKII, modest elevations in postsynaptic Ca^2+^ will lead to preferential activation of calcineurin over the CaMKII, resulting in LTD. In contrast, upon high-frequency stimulation, the Ca^2+^ concentration is higher, resulting in activation of protein kinases, such as CaMKII. This, in turn, promotes the phosphorylation of α-amino-3-hydroxy-5-methyl-4-isoxazole propionic acid receptors (AMPARs), as well as the exocytosis of AMPARs into the postsynaptic membrane.

**Figure 4:**
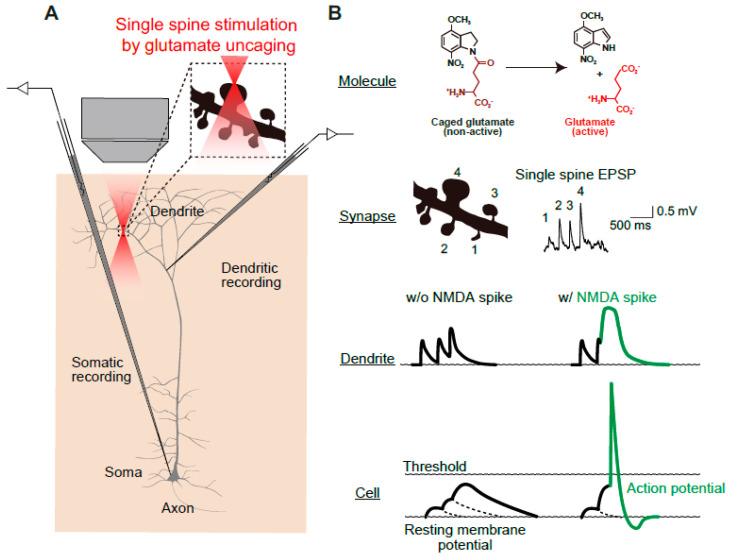
Schematics of multi-scale analyses of *N*-methyl-D-aspartate-type glutamate (NMDA)-dependent synaptic and somatic events. (**A**) Simultaneous measurement of dendrite and somatic membrane potentials during precise stimulation of identified spines by two-photon uncaging of 4-methoxy-7-nitroindolinyl (MNI)-glutamate. (**B**) Molecular structures of MNI-glutamate. The glutamate uncaging relies on conversion of an inert caged compound (MNI-glutamate) into glutamate by two-photon laser radiation. The spatial resolution of the two-photon laser is sufficiently fine to allow stimulation of a single spine (lateral and axial full-width, half-maximal diameters of 0.29 and 0.89 μm, respectively [16]). Glutamate uncaging evokes a single-spine EPSP response, which is an index of the synaptic weight of each spine. Dendritic and whole-cell patch-clamp recordings provide the dendritic and somatic membrane potential, respectively. In the presence of an NMDA spike, which is evoked by simultaneous inputs into multiple spines, an action potential is elicited. Images are adapted from [17] with slight modification.

Each neuron receives and integrates thousands of glutamatergic synaptic inputs. The ionotropic glutamate receptors mainly belong to two groups. The first is α-amino-3-hydroxy-5-methyl-4-isoxazole propionic acid receptors (AMPARs), which are mainly permeable to Na^+^ and K^+^, mediate fast synaptic transmission (Figure 2). Alterations in the number of AMPARs at the post-synapse are among the major mechanisms of synaptic plasticity, in which the insertion and removal of AMPARs take place during long-term potentiation (LTP) and long-term depression (LTD), respectively (Figure 3) [5]. The second type of ionotropic glutamate receptor is the NMDAR, which consists of tetramers of two GluN1 and two GluN2 subunits that bind D-serine and glutamate, respectively (Figure 2B) [6,7,8]. A well-known feature of NMDARs is their strong, voltage-dependent block by external Mg^2+^ at resting potential, as well as their permeability to Ca^2+^, which acts as a second messenger and activates various calcium-dependent proteins [9], which are crucial for synaptic plasticity (Figure 3). Consistent with this, pharmacological or genetic ablation of NMDARs or CaMKII in the hippocampus results in impairment of LTP and spatial learning [9,10]. The third characteristic feature of NMDARs is their regenerative electrogenesis, which is defined as a positive-feedback inward current with additional depolarization. Consequently, simultaneous activation of NMDARs via clustered synaptic inputs, involving the activation of at least eight spines, can generate N-methyl-D-aspartate-type glutamate (NMDA) spikes (Figure 4B), which in turn can trigger a dendritic Ca^2+^ spike in the proximal dendrite. Thus, the neuron has three gating thresholds [11,12]: the NMDA spike at the distal tuft branches, which receive synchronous activation from clustered neighboring glutamatergic synapses [13]; multiple NMDA spikes among different distal dendritic branches, which trigger dendritic Ca^2+^ spikes through voltage-dependent Ca^2+^ channels near the main bifurcation [14]; and ultimately, the generation of an action potential [15]. In this regard, neurons can be thought of as implementing a “synaptic democracy”, in which the incoming signals of the bulk of synaptic inputs are integrated and tallied, and collectively contribute to firing; the NMDAR is a crucial component of this process. Furthermore, a recent study further confirmed the correlation between NMDA spikes and animal behavior [13]. Together, these observations suggest that multi-scale functions of NMDARs interconnect synaptic inputs, dendritic spikes, somatic action potential discharges in neurons, and eventually behavioral outputs (Figure 4).

## 3. Evidence of NMDAR Dysregulation from Clinical Studies of Schizophrenia

The involvement of NMDAR dysfunction in the pathophysiology of schizophrenia was attributed initially to the observation that non-competitive NMDAR antagonists, such as phencyclidine (PCP) and ketamine (Figure 2B), induce a behavioral psychopathology similar to that of schizophrenia patients in healthy subjects [18]. The efficacy of PCP in inducing all aspects of the symptomatology of schizophrenia is unparalleled, and even experienced psychiatrists misdiagnose chronic PCP abusers as schizophrenic [19]. Consistent with this, systemic administration of PCP to rodents and non-human primates induces schizophrenia-related behavioral alternations, in association with initial hyperactivity of prefrontal pyramidal neurons, followed by a delayed depression of activity [20]. The main function of a neuron, in general, is to receive and integrate synaptic inputs and then generate a sequence of action potentials or “spike train”. What makes the pyramidal neurons special is that they are “projection neurons” that send their axons over long distances to connect different brain regions (brain-wide circuit scale). Thus, dysregulation of this cell type can have profound effects on brain-wide neural circuits. NMDAR dysfunction in a subset of schizophrenia can be related to the kynurenic acid (KYNA) dysregulation. Increased KYNA concentrations have been consistently reported in the cerebrospinal fluid and the postmortem brains of schizophrenic patients [21]. High levels of KYNA in the prefrontal cortex of patients with schizophrenia can be due to reduced activity of kynurenine 3-monooxygenase (KMO), because reduction in KMO expression and its enzyme activity have been reported in the patient’s brain [21]. At low concentrations, KYNA acts as a non-antagonistic inhibitor of the α7 nicotinic acetylcholine receptor (α7nAch-R), and at high concentrations, it acts as an antagonistic inhibitor of NMDAR (Figure 2B), reducing glutamate and acetylcholine transmission, which may lead to cognitive decline.

Another piece of evidence implicating NMDAR dysfunction in schizophrenia was obtained from an examination of anti-NMDAR encephalitis, an acute inflammation (swelling) of the brain. The symptoms of anti-NMDAR encephalitis resemble the severe psychotic symptoms of schizophrenia (hallucinations and delusion) [22]. The enthusiasm about this discovery of anti-NMDAR encephalitis is due to its unambiguous etiology: this subtype of psychosis can be segregated from schizophrenia, but more importantly, it can be treated with immunotherapy and surgical removal of the antibody-producing tumor. NMDAR encephalitis is associated with antibodies against the extracellular epitopes of the GluN1A subunit (Figure 2B) [23]. According to a meta-analysis of human studies, the level of NMDAR antibodies is significantly higher in patients with first-episode psychosis than in healthy controls, suggesting that the pathogenic potential of NMDAR antibodies is not restricted to anti-NMDAR encephalitis, but is also involved in at least a subset of general schizophrenia [24]. Thus, our understanding of how neuronal circuits are altered in NMDAR encephalitis can be applied, at least to some extent, to genuine schizophrenia. This raises the question of how autoantibodies for NMDARs elicit schizophrenia-like behaviors. NMDAR antibodies are thought to cause antibody-mediated cross-linking of NMDARs, followed by internalization of the receptors in both excitatory and inhibitory neurons [23]. The ultimate consequence is an increase in the imbalance between excitation and inhibition, which could in turn increase the excitability of pyramidal neurons. The relative increase in excitatory transmission in anti-NMDAR encephalitis may be consistent with the results of proton magnetic resonance spectroscopy (MRS) studies. MRS allows non-invasive quantification of both stationary and dynamic information of the brain, and ^1^H-MRS is used for measuring metabolites related to neurotransmission, such as glutamate and glutamine. A longitudinal, 7-Tesla MRS study with drug-naïve or minimally medicated first-episode schizophrenia patients revealed that higher glutamate in the dorsal anterior cingulate cortex (dACC) was associated with more severe functional impairment in the first episode [25]. Studies of chronic schizophrenia patients have reported a negative correlation between dACC glutamatergic neurometabolite levels (Glx, the sum of glutamate and glutamine) and cortical thickness, implying that glutamate-mediated excitotoxicity is one of the mechanisms underlying the structural compromises seen in treatment-resistant schizophrenia [26]. A meta-analysis of ^1^H-MRS studies revealed that Glx was significantly higher in the frontal lobe of individuals who were genetically at high risk for schizophrenia than in healthy controls [27].

An additional line of evidence is provided by neuropathological studies from autopsied brains of individuals with schizophrenia. One of the most consistently observed postmortem alterations in the brains of schizophrenia patients is the reduction in dendritic spine density [28,29,30]. This reduction is observed in layer III pyramidal neurons of the PFC, but not in layer V or VI, nor in layer III of the primary visual cortex [31], suggesting that the synaptic pathology of schizophrenia takes place in specific neuronal circuits, rather than throughout the entire brain. Immunohistochemistry experiments have revealed that the level of the postsynaptic marker spinophilin, a protein that is highly enriched in dendritic spines, is reduced in schizophrenic patients, providing complementary evidence for the reduction in spine density [32]. The reduction in dendritic spines is likely to be a result of the illness itself rather than of antipsychotics, which do not decrease dendritic spine density [33]. Interestingly, a within-subject analysis revealed a highly significant correlation between antemortem prefrontal cognitive deterioration and loss of *GluN1* mRNA in the postmortem temporal cortex of patients with schizophrenia [34]. Expression of *GluN1* mRNA does not correlate with age or duration of the disorder, suggesting that the reduction may not be due to atrophic changes in neural circuitry resulting from prolonged hospitalization or long-term exposure to antipsychotics. The decrease in synaptic markers in the postmortem brain and the increase in glutamatergic markers observed in MRS studies of the early stage of schizophrenia patients seem contradictory. This could be due to changes occurring during its progression; glutamatergic transmission is pathologically exacerbated around the onset of the disorder, and this is eventually followed by a reduction and dysregulation of neurotransmission. Alternatively, the decrease in spine density could enhance neuronal excitability via unknown mechanisms. To identify the precise mechanism of NMDAR dysfunction in neural circuits in schizophrenia-related conditions from a multi-scale perspective, we will discuss in the next sections the use of animal models and advanced imaging and optogenetic techniques.

## 4. Utilization of Animal Models to Gain Insight into NMDAR-Dependent Synaptic Pathology at the Circuit Level

The most important limitation of studies of psychiatric disorders are the ethical challenges associated with studying the human brain in vivo. Even when the most advanced ultra-high-field magnetic resonance imaging methods are used [35], it is extremely difficult to visualize circuit pathophysiology at synaptic resolution. Postmortem brain research is a useful strategy, but samples from the postmortem brain are subject to various artifacts related to medication, the cause of death, agonal state, and the length of the interval from death to post-mortem examination [36]. State-of-art techniques, such as iPS cells and their organoids (mini-brains), are still insufficient to faithfully recapitulate the complexity of the human brain [37]. Hence, although the rodent species does not share all aspects of the human brain, humans and rodent brains nonetheless share evolutionarily conserved structural and functional processes. In particular, relatively primitive cognitive domains, such as memory, fear, and impulsivity, can be extrapolated using rodent models. Although no animal model exhibits all of the categories of schizophrenia symptoms (positive symptoms, negative symptoms, and cognitive deficits), we can evaluate and compare NMDAR-related schizophrenia models to extract some commonly shared phenotypes, and to understand the neurobiological bases of schizophrenia.

Several genetically engineered mouse models were designed by inducing mutations into NMDAR subunits. Mohn et al. generated mouse strains that express the GluN1 subunit at <10% of the normal level, resulting in behavioral abnormalities that are qualitatively and quantitatively similar to mice treated with the NMDAR antagonist MK801. The observed phenotypes include locomotor hyperactivity, stereotypy, deficits in social and sexual interactions, and reduced performance in spatial working memory tasks and sensorimotor gating [38]. Deletion of GluN1 subunits in 40–50% of cortical and hippocampal interneurons in early postnatal development provided further evidence for the cell type-specific roles of NMDARs in schizophrenia [39]. This model exhibited a broad variety of behavioral abnormalities relevant to schizophrenia, including elevated anxiety-like behaviors in the open field and plus-maze tests, as well as impaired prepulse inhibition (PPI). Post-adolescent deletion of GluN1 did not result in such behavioral abnormalities, suggesting the importance of early postnatal inhibition of NMDAR activity in inhibitory neurons as the contributory factor. In other words, these findings suggest that disinhibition of excitatory neurons and reduced neuronal synchrony during this period are relevant to the developmental origin of schizophrenia.

Calcineurin is one of the most important downstream molecules of NMDAR signaling (Figure 3), and is encoded by a schizophrenia susceptibility gene. Calcineurin is a Ca^2+^- and calmodulin-dependent serine/threonine protein phosphatase that is predominantly identified within the postsynaptic densities and soma of neurons [40]. Genetic association studies of schizophrenia have revealed a significant link to variations in the gene encoding the calcineurin A γ subunit (PPP3CC) gene [41] and other calcineurin-related genes [42]. Tonegawa and colleagues generated a forebrain-specific, conditional calcineurin knockout (calcineurin cKD) mouse model [43]; this model also exhibits abnormalities, such as elevated locomotor activity, impaired social interaction, and severe hippocampus-dependent working memory deficit [44]. Electrophysiological analysis of the hippocampus have indicated that calcineurin cKD mice are deficient in NMDAR-dependent LTD [43]. Consistent with this, this model exhibited a shift of the dendritic spine volume into a larger fraction [45], possibly owing to the deficit in LTD. The selective loss of small spines in the calcineurin cKD is consistent with postmortem studies of patients with schizophrenia, in whom the decrease in the number of spines is only observed in smaller spines (<0.15 μm^3^) [46]. Currently, however, we lack mechanistic insights into how the shift of spine diameter could shape the basis of altered circuitry in schizophrenia.

**Figure 5:**
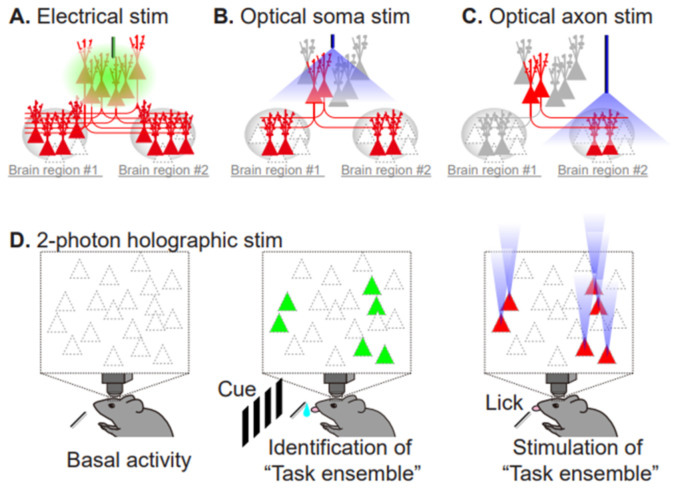
Comparison of electrical stimulation and the currently available repertoire of optical stimulation techniques. (**A**) Electrical pulses reach the targeted neuron and evoke neuronal firing, but also indiscriminately impact adjacent neurons and their projecting neurons (red). (**B**) Optical stimulation specifically increases the firing rate of opsin-expressing neurons (red). (**C**) Axonal stimulation enables projection-specific activation within the brain region of interest. (**D**) Two-photon, holographic optogenetics allows simultaneous monitoring and stimulation of task-related neurons. Task-performing animals are subjected to in vivo, 2-photon imaging to monitor neuronal activity, using a calcium indicator (green, active cells during the task). Then, photostimulation of the identified subset of neurons (red) recapitulates the task-related neuronal ensemble activity pattern, evoking the task performance without the cue.

Among a number of susceptibility genes involved in schizophrenia, the *DISC1* (disrupted in schizophrenia 1) gene is one of the best characterized. DISC1 was originally identified in a large Scottish family with a chromosomal translocation at t(1;11), which has a robust impact in this pedigree (logarithm of the odds (LOD) ratio = 7, equivalent to 10^7^:1 odds that the translocation is linked to psychiatric disorders). Although genome-wide analyses for schizophrenia failed to provide support for *DISC1* itself as a common risk gene for schizophrenia [47], a large set of DISC1-interacting proteins (DISC1 interactome) have disruptive variants that are significantly associated with low cognitive ability in schizophrenia [48]. At the synapse, DISC1 works as a negative regulator of Rac1-GEF Kalirin-7, which acts downstream of NMDAR activation, and knockdown of DISC1 results in the deterioration of spine morphology [49]. Because the overall outcomes elicited by this translocation are thought to be equivalent to haploinsufficiency [50], we will focus on knockouts and knockdowns of DISC1. Conventional knockout mice exhibit various behavioral abnormalities, including elevated anxiety, higher impulsivity, impairment of PPI, increase in context-dependent, conditioned fear freezing, and methamphetamine-induced hyperactivity that is indicative of dopaminergic hyperexcitability [51]. The mouse model that most closely mimics the Scottish t(1;11) carriers was derived from the Gogos laboratory [52]. Their research group generated mutant mice carrying two termination codons in exon 7, resulting in the elimination of full-length DISC1 and very low expression of the truncated DISC1 protein. Cognitive tests, such as the two-choice, delayed, non-match to position task, revealed that this model has a selective impairment in working memory. These mice also exhibited a significant reduction in the number of spines in granule cells in the hippocampus. Taken together, these findings indicate that proper expression of DISC1 is required for neuronal circuitry and cognitive functions. The most important question is whether these observations reflect causal connections, or merely reflect a correlation, or even worse, compensation that is a secondary consequence of schizophrenia. Necessity and sufficiency establish causation. Biologically, if manipulation of a factor interferes with the phenomenon under study, then this shows that the factor is necessary for the appearance of the phenomenon. If artificial activation of a factor elicits the phenomenon, the factor is considered as sufficient for the phenomenon in biology. How can we directly identify psychiatric pathology from the standpoint of multi-scale causality? In other words, how do disruptive mutations in susceptibility molecules alter synaptic transmission and plasticity in neuronal circuits, eventually resulting in behavioral manifestations? In this regard, optogenetics, which relies on the precise targeting and optical control of opsin proteins, can achieve even cell-type and pathway-specific activation, opening a completely new way to establish necessity and sufficiency for the elucidation of how neuronal networks work, and how they relate to behaviors, while also providing important information for the neural coding and computation of neural circuits. Optogenetics uses light to manipulate molecular and cellular events in targeted cells of living animals in vivo. This method is a fruitful technological fusion of optics and genetic engineering that maximizes the advantage of each discipline: millisecond-scale and fine temporal regulation of optical control, and specific gene expression and trafficking of the gene product with subcellular precision. For example, channelrhodopsin-2 is a light-activated cation channel that enables control of the membrane potential of targeted neurons, driving it above the action potential threshold [53]. Thus, it is possible to control the neural activity of opsin-expressing neurons using blue light with millisecond temporal precision, which cannot be achieved by electrode-based stimulation (Figure 5A,B). In addition, photo-inhibitory membrane proteins have been engineered [54]. Consequently, optical activation, silencing, and (de)synchronization of neuronal activity have become possible, providing evidence of how neuronal networks work and how they relate to behaviors [55]. To date, optogenetics has been extensively applied to studies of neuropsychiatric disorders, such as depression; anxiety, reward, and motivation in laboratory animals; and dysfunctions in neuronal circuits related to depression [56], anxiety, addiction [57], decision making [58], and compulsive behaviors [59]. Not only can entire cellular behaviors be controlled by somatic activation, but input-specific optogenetic interrogation in a specific brain region can also be achieved by specific photoactivation of axon terminals (Figure 5C). One of the first studies using this technique revealed that optogenetic inhibition of connections between the central amygdala and basolateral amygdala induced an innate anxiety behavior, whereas direct stimulation of basolateral amygdala somata did not [60]. This study has amply demonstrated that circuit activity as a whole comprises distinct sets of synaptic inputs from different brain regions, whose plasticity leads to the acquisition of a specific behavior. Furthermore, two-photon, holographic optogenetics allows manipulation of task-related neurons in the animal, allowing evocation of the learned, visually-guided task behavior (Figure 5D) [61].

**Figure 6:**
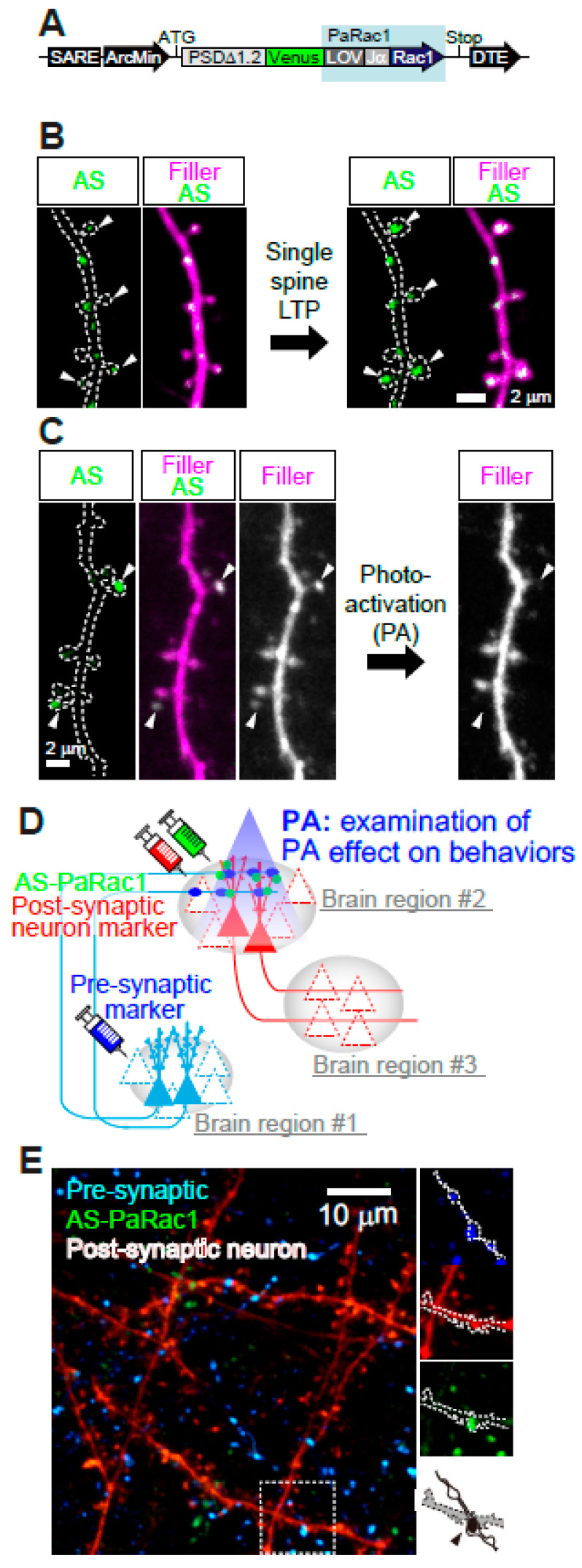
Multi-scale labeling and manipulation of NMDA-dependent synaptic and circuit plasticity. (**A**) AS-PaRac1 is a fusion of five genes: a partial promoter of *Arc*, the N-terminal of *PSD-95*, *Venus* (green fluorescence), the light–oxygen–voltage (LOV) domain of *phototropin*, *Rac1*, and the dendritic targeting element (DTE) from the 3’ UTR of the *Arc* mRNA. Upon blue light radiation, excitation of the flavin mononucleotide induces a conformational change in the LOV domain, followed by dissociation and unfolding of the LOV domain from the constitutively active form of Rac1. (**B**) Specific accumulation of AS-PaRac1 in potentiated spines. Single-spine, long-LTP protocol evoked by glutamate uncaging induces accumulation of AS-PaRac1 in the stimulated spines (arrowheads) of pyramidal neurons. (**C**) Selective shrinkage of AS-PaRac1-positive spines by photoactivation (PA). Robust shrinkage (arrowheads) is observed, whereas AS-PaRac1-negative spines are not affected by PA, despite being located adjacent to AS-PaRac1-positive spines. (**D**) Possible experimental designs for multi-scale functional imaging. NMDAR-dependent expression of the presynaptic marker (blue), AS-PaRac1 (green), and postsynaptic neuron marker (red) can visualize spine potentiation. (**E**) Representative two-photon microscopy for multi-scale functional imaging in the primary motor cortex of a mouse. Images are adapted, with permission, from [62,63].

Although optogenetics is a very powerful tool for controlling the action potentials of neurons, it cannot manipulate individual synapses. Given that distinct subsets of synapses are activated in different contexts [62], photo-manipulation at the level of the synapse, rather than the entire cell, would provide a complementary tool for elucidating the causal role of synaptic function. Toward this goal, which we have named “synaptic optogenetics,” we developed a novel synaptic optoprobe, AS-PaRac1 (activated synapse targeting photoactivatable Rac1) (Figure 6A) [63]. This photoprobe has two characteristic features. First, it can specifically label recently potentiated (enlarged or newly formed) spines (Figure 6B). The expression of this photoprobe is regulated by synaptic activity responsive elements (SARE) in the *Arc* gene promoter, the activity of which is dependent on synaptic Ca^2+^ influx through NMDARs; which allows NMDAR-dependent expression of AS-PaRac1. Second, AS-PaRac1 induces the selective shrinkage of AS-PaRac1-containing spines upon blue light radiation (Figure 6C). Thus, researchers can not only label potentiated spines with AS-PaRac1, but also induce specific shrinkage of potentiated spines using blue light. In experiments, learning-promoted spine enlargement and new spine generation, as well as 70–80% of potentiated spines, were successfully labeled by AS-PaRac1. Blue light radiation induced the shrinkage of the learning-related, AS-PaRac1–containing spines, resulting in disruption of the acquired learning. This experiment provided the first proof of concept demonstrating that plasticity of dendritic spines is an important mechanism underlying behavior. In other words, these findings have revealed multi-scale causality between NMDAR-dependent synaptic plasticity and behaviors. Furthermore, AS-PaRac1 can be applied to Hebbian plasticity, an important cellular mechanism that is often used to model activity-dependent reorganization of neuronal selectivity in various aspects of learning [64]. Such plasticity needs to increase postsynaptic firing rates to high levels, so that the neuron in a selected cell ensemble becomes more responsive to others. Because induction of Hebbian plasticity requires the correlated firing of presynaptic and postsynaptic neurons, simultaneous visualization of both pre- and postsynaptic activation by whole-brain imaging at single-cell resolution would provide new insight into circuit plasticity. Among the activity-dependent genes, the activation of synaptic activity responsive elements (SARE) in the *Arc* gene is dependent on synaptic Ca^2+^ influx through NMDARs. Thus, using a SARE-Arc promoter, NMDAR-dependent expression of the presynaptic marker AS-PaRac1, and a postsynaptic neuron marker, we can visualize spine potentiation (synaptic scale) and circuit activation, which consists of the presynaptic and postsynaptic neurons among multiple brain regions. Via blue light-induced shrinkage of labeled spines and subsequent assessment of behavioral manifestations, this method can extract the neurocircuits that play causal roles in NMDAR-related behaviors (Figure 6D). Furthermore, retrograde tracing of the presynaptic neuron together with anterograde tracing of the postsynaptic neuron could provide NMDA-dependent circuit plasticity among several brain regions (e.g., #1–3 in Figure 6D). Although these experiments alone did not contribute to the understanding of human psychiatric disorders, visualization and manipulation of NMDA-dependent synaptic and circuit plasticity in various validated animal models could provide mechanistic insight with single synapse resolution. Furthermore, the use of animals in research is also essential for the development of new drugs and treatments. Monitoring NMDA-dependent synaptic and circuit plasticity and comparing the results with behavioral outcomes in animals could be an intermediate micro-endophenotype to validate the therapeutic effectiveness of new drugs. Until techniques that allow synaptic resolution imaging and invasive experiments in the human brain become available, the bidirectional cycle between animal model studies, employing well-designed multi-scale imaging and manipulative experiments, and clinical studies with human samples will be necessary (Figure 1).

## 5. Closing Remarks/Conclusions

In this review, we examined the involvement of synaptic disturbances, mainly those generated by NMDARs, in the pathophysiology of schizophrenia. Despite the accumulation of evidence supporting NMDAR-mediated synaptic dysfunction in schizophrenia, we still do not know in which synapses, in which dendrites, in how many neurons, and in which brain regions, even in model animals, NMDAR function is altered. This is one of the reasons why therapeutic strategies aimed at modulating NMDAR-related transmission have unfortunately remained unsuccessful. Recent technical developments in large-scale imaging and optical manipulation of synaptic plasticity afford an unprecedented opportunity to link synaptic- and circuit-scale plasticity brain-wide to behavioral outputs. Future extensions and combinations of these cutting-edge techniques will enable coherent accounts to be proposed for the multi-scale mechanisms of learning from the synapse, to the cell/circuit, to the behavioral level. Such multi-scale causal understanding is necessary for establishing circuit-centric therapeutics as a complement to current molecular (and chemistry)-based drug designs.
